# Severe Iatrogenic Calcinosis Cutis From Extravasated Calcium Gluconate

**DOI:** 10.7759/cureus.9712

**Published:** 2020-08-13

**Authors:** Brad E Rumancik, Sahand Rahnama-Moghadam

**Affiliations:** 1 Dermatology, Indiana University School of Medicine, Indianapolis, USA

**Keywords:** iatrogenic calcinosis cutis, dystrophic calcification, extravasation, calcium gluconate, calcium chloride

## Abstract

Iatrogenic calcinosis cutis occurs when insoluble calcium salts deposit in cutaneous and subcutaneous tissue. Iatrogenic calcinosis cutis is a rare complication from a variety of medical interventions, most commonly due to extravasated intravenous calcium-containing solutions. We present a severe case of iatrogenic calcinosis cutis in a patient with end-stage renal disease and an elevated serum calcium-phosphate product. Iatrogenic calcinosis cutis has a wide range of clinical presentations. Either subclinical or clinically noticeable extravasations may cause mild to severe calcinosis cutis. Patients with increased serum calcium and phosphate may be at increased risk of iatrogenic calcinosis cutis. Treatment options include conservative, pharmacologic, or surgical management.

## Introduction

Calcinosis cutis is defined as insoluble calcium salt deposition in cutaneous and subcutaneous tissue [1,2]. There are five subtypes of calcinosis cutis based on etiology: dystrophic, metastatic, calciphylaxis, idiopathic, and iatrogenic. Dystrophic calcinosis cutis is due to local tissue damage secondary to trauma, infection, neoplastic processes, or a variety of connective tissue disorders. Metastatic calcinosis cutis, a rare manifestation, occurs secondary to systemic calcium and phosphate abnormalities, classically occurring in chronic renal failure. Calciphylaxis, a related yet distinct entity from metastatic calcinosis cutis, occurs when small and medium-sized blood vessels in the dermis and subcutaneous fat calcify. Idiopathic calcinosis cutis occurs without known local tissue injury or metabolic disorders [1,2]. Lastly, iatrogenic calcinosis cutis most commonly occurs from extravasation of calcium or phosphate-containing solutions [3]. Iatrogenic calcinosis cutis has been reported in patients undergoing electroencephalography or electromyography with prolonged periods of exposure to saturated calcium-containing electrode paste [4-6]. Iatrogenic calcinosis cutis has also occurred in patients receiving subcutaneous injections of para-aminosalicylic acid, solid organ transplant recipients, and neonates receiving numerous heel venipuncture sticks [1,2]. We present a severe case of iatrogenic calcinosis cutis after extravasation of calcium gluconate solution.

## Case presentation

A 55-year-old woman with no underlying connective tissue disease and a past medical history of hemodialysis-dependent end-stage renal disease due to type 2 diabetes mellitus and hypertension was hospitalized for left lower extremity cellulitis who requested inpatient removal of a right forearm plaque. The lesion location corresponded with the site of an extravasated calcium gluconate peripheral intravenous infusion, which was administered 13 days prior during a previous hospitalization requiring urgent hemodialysis to treat hyperkalemia. The patient described an initial burning sensation during the extravasation, but the subsequent calcification, which developed over a few days, was insensate. 

**Figure 1:**
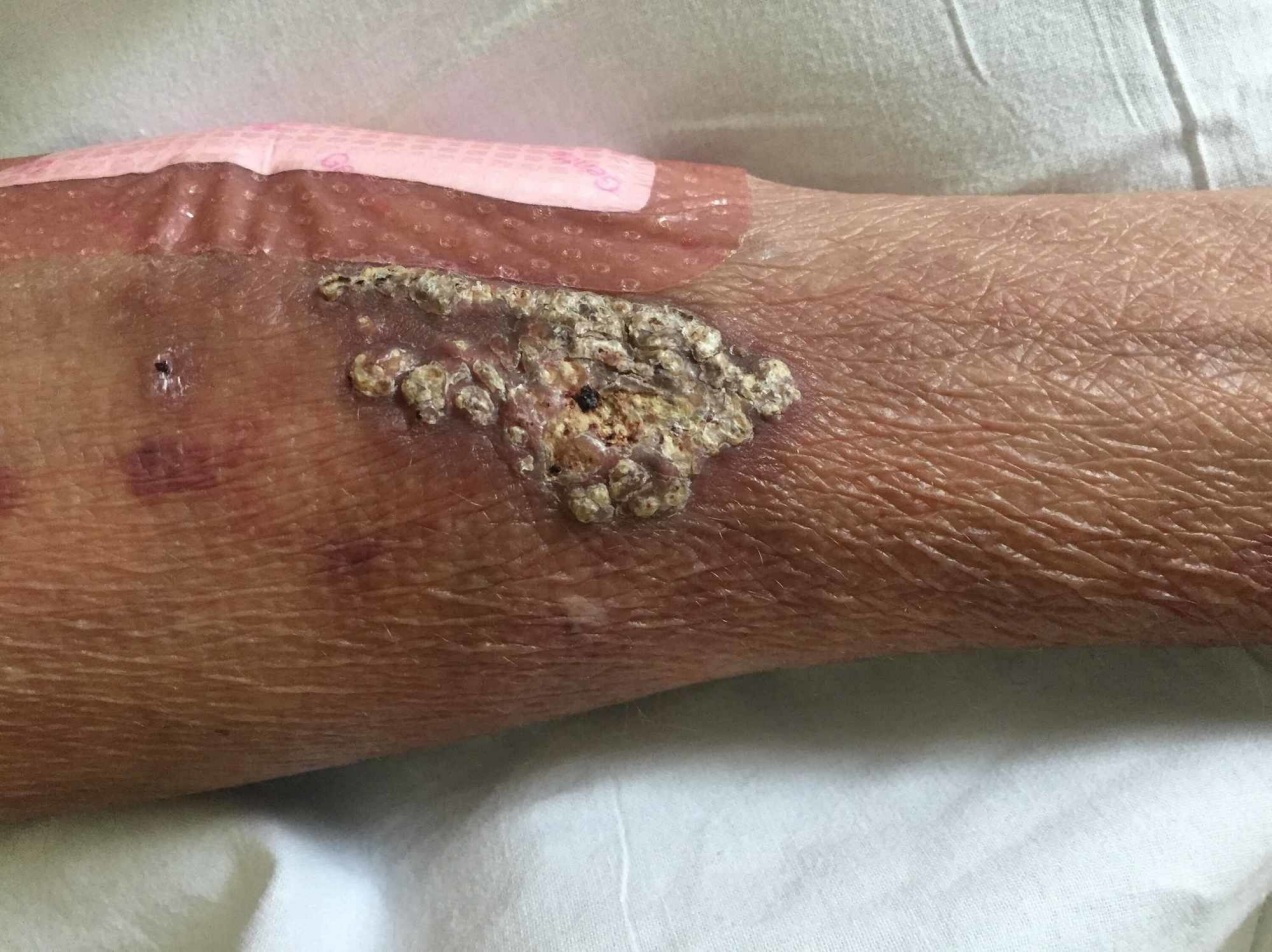
Indurated plaque on the right flexor forearm at the site of extravasated calcium gluconate infusion.

On examination, a yellow-white, rock-hard indurated plaque measuring approximately 4 × 3 cm with overt mineral deposits was found on the right distal flexor forearm (Figure 1). The bandage next to the lesion in question is to remedy an unrelated trauma-induced injury. At the time of physical examination, her serum corrected calcium level was 10.1 mg/dl (normal range: 8.5-10.1 mg/dl) and phosphate level was 7.6 mg/dl (normal range: 2.5-4.9 mg/dl). A clinical diagnosis of iatrogenic calcinosis cutis was made based on the infusion history and the localized calcification. Outpatient surgical intervention was chosen given her severe presentation; however, the patient expired from a fatal arrhythmia while waiting for the procedure.

## Discussion

Extravasation of any calcium-containing peripheral infusion can cause iatrogenic calcinosis cutis [2,3,7]. Extravasations often lead to abrupt symptoms of erythema, tenderness, induration, and edema; however, subclinical extravasations may lack these initial symptoms and still present with calcinosis. Calcinosis typically develops within two weeks as yellow-white papules, plaques, or nodules with possible tissue necrosis or ulceration. Calcinosis can occur despite removal of the intravenous line suspected of extravasation [3,7].

Subcutaneous injection of calcium-containing solutions in rats led to formation of mineralized plaques with a total calcium content 100 times greater than the amount of calcium infused [8]. Therefore, the hypothesized pathogenesis of iatrogenic calcinosis cutis is mineralization of calcium derived from extravasated calcium solutions and cellular release of calcium from local tissue damage [3,8-10]. This mechanism may explain the development of iatrogenic calcinosis cutis in subclinical extravasations and the variability in degree of calcinosis among patients. An elevated calcium-phosphate product is most associated with metastatic calcinosis cutis and calciphylaxis [1,2]. However, authors have suggested an elevated calcium-phosphate product may increase susceptibility to iatrogenic calcinosis cutis, which may have contributed to our patient’s severe calcinosis [9]. Lastly, the presence of collagen, which facilitates calcium phosphate deposition, is believed to be a critical factor in the pathogenesis of iatrogenic calcinosis cutis. Histologic samples of affected tissue show calcium deposits in close proximity to degenerated collagen fibers within the dermis [3,10].

Prevention of extravasation is the most important mitigating factor in developing iatrogenic calcinosis cutis. Use of a central line or diluted calcium and phosphate solutions may reduce rates of iatrogenic calcinosis cutis [9]. Calcium chloride, an inorganic calcium salt, has a higher risk of skin necrosis and adverse effects from extravasation compared to calcium gluconate, an organic calcium salt [11,12]. Theories suggest this heightened risk from calcium chloride is most related to increased dissociation tendency and thus elevated local ionized calcium concentration [11,12]. Local ionized calcium concentration appears to be of greater significance than other factors such as pH or osmolarity [12,13].

Immediate management of clinically recognized extravasations is not well defined. Intralesional triamcinolone acetonide and hyaluronidase was shown to reduce severity and rate of development of calcinosis cutis following extravasated calcium-containing solutions in rabbit models [14,15]. However, there is scant human evidence for these therapies, and conservative measures, such as elevation of the extravasation site and cold compresses, predominate in current practice [11,16].

There is no standardized treatment for iatrogenic calcinosis cutis once it occurs [16,17]. Treatment regimens are largely derived from case reports. Options include conservative, pharmacologic, or surgical management. Typically, less severe cases of iatrogenic calcinosis cutis resolve in two to six months with conservative local wound care alone [3]. Sodium thiosulfate, a medication utilized systemically for calciphylaxis or locally for dystrophic calcification, has cation chelating properties allowing for soluble calcium thiosulfate complex formation [9]. Two separate case reports, including a six-year-old boy and a sixty-year-old man with iatrogenic calcinosis cutis, describe topical sodium thiosulfate treatment leading to complete resolution of calcinosis cutis as described in Table 1 [9,18]. There are no other effective pharmacologic therapies studied specifically for iatrogenic calcinosis cutis. Other pharmacologic options are derived from treating different forms of calcinosis cutis, including bisphosphonates, diltiazem, warfarin, minocycline, ceftriaxone, aluminum hydroxide, probenecid, intralesional corticosteroids, and intravenous immunoglobulin [9,11,17]. Severe cases, such as our case patient, require surgical intervention [2,3].

**Table 1:** Comparison of two published cases of iatrogenic calcinosis cutis successfully treated with topical sodium thiosulfate

Author, year of publication	García-García et al., 2017 [9]	Abbott et al., 2020 [18]
Patient	Six-year-old boy	Sixty-year-old man
Past medical history	Permanent hypoparathyroidism from prophylactic thyroidectomy due to carrier status of RET proto-oncogene and family history of multiple endocrine neoplasia type 2	Chronic obstructive pulmonary disease on tacrolimus for right-sided lung transplant three months prior to case presentation date
Reason for presentation	Severe hypocalcemia	Cardiac arrest secondary to a complicated bronchoscopy
Inciting agent	Intravenous calcium gluconate	Intravenous calcium chloride
Location of infusion and initial signs and symptoms	Left antecubital fossa infusion led to phlebitis prompting removal and placement in right antecubital fossa which also led to phlebitis	Left dorsal hand. No initial signs or symptoms as patient required immediate intubation due to cardiac arrest
Time to notice iatrogenic calcinosis cutis	Bilateral calcinosis occurred within a few days of infusion	Two weeks following infusion after patient was extubated
Description of iatrogenic calcinosis cutis lesion	On bilateral antecubital fossae, multiple erythematous nodules with extrusion of yellow calcified substance	On left dorsal hand, 4 x 3 cm white sclerotic plaque
Symptoms at time of recognizing iatrogenic calcinosis cutis	Severe motility impairment and pain	Asymptomatic
Treatment	Compounded 10% topical sodium thiosulfate in a water-in-oil emulsion cold cream applied every morning and night under occlusion	Compounded 10% topical sodium thiosulfate lotion applied twice daily under occlusion
Therapeutic course	After three months of treatment: dramatic improvement in skin lesions and motility. After six months of treatment: normal skin appearance and motility	After one month of treatment: 95% reduction in plaque size. After two months of treatment: 99% plaque resolution. At four-month assessment (after an additional one to two weeks of focal applications to residual white spots): complete resolution
Adverse effects	None reported	None reported

## Conclusions

Iatrogenic calcinosis cutis is an uncommon manifestation most commonly occurring after extravasation of calcium-containing solutions. Clinicians should be aware of the variable time course and severity resulting from either clinically insignificant or noticeable extravasations. Treatment should be considered immediately after extravasations and upon development of calcinosis cutis. However, no agreed upon treatment protocol for these clinical scenarios exists.
